# Assessing the Viscoelasticity of Photopolymer Nanowires Using a Three-Parameter Solid Model for Bending Recovery Motion

**DOI:** 10.3390/nano11112961

**Published:** 2021-11-04

**Authors:** Jana Kubacková, Cyril Slabý, Denis Horvath, Andrej Hovan, Gergely T. Iványi, Gaszton Vizsnyiczai, Lóránd Kelemen, Gabriel Žoldák, Zoltán Tomori, Gregor Bánó

**Affiliations:** 1 Department of Biophysics, Institute of Experimental Physics SAS, Watsonova 47, 040 01 Košice, Slovakia; kubackova@saske.sk (J.K.); tomori@saske.sk (Z.T.); 2 Department of Biophysics, Faculty of Science, P. J. Šafárik University, Jesenná 5, 041 54 Košice, Slovakia; cyril.slaby@student.upjs.sk (C.S.); andrej.hovan@upjs.sk (A.H.); 3 Center for Interdisciplinary Biosciences, Technology and Innovation Park, P. J. Šafárik University, Jesenná 5, 041 54 Košice, Slovakia; denis.horvath@upjs.sk (D.H.); gabriel.zoldak@upjs.sk (G.Ž.); 4 Faculty of Science and Informatics, University of Szeged, Dugonics Square 13, 6720 Szeged, Hungary; itgergo@gmail.com; 5 Biological Research Centre, Institute of Biophysics, Eötvös Loránd Research Network (ELKH), Temesvári krt. 62, 6726 Szeged, Hungary; vizsnyiczai.gaszton@brc.hu (G.V.); kelemen.lorand@brc.hu (L.K.)

**Keywords:** two-photon polymerization, nanowire, viscoelastic material, standard linear solid

## Abstract

Photopolymer nanowires prepared by two-photon polymerization direct laser writing (TPP-DLW) are the building blocks of many microstructure systems. These nanowires possess viscoelastic characteristics that define their deformations under applied forces when operated in a dynamic regime. A simple mechanical model was previously used to describe the bending recovery motion of deflected nanowire cantilevers in Newtonian liquids. The inverse problem is targeted in this work; the experimental observations are used to determine the nanowire physical characteristics. Most importantly, based on the linear three-parameter solid model, we derive explicit formulas to calculate the viscoelastic material parameters. It is shown that the effective elastic modulus of the studied nanowires is two orders of magnitude lower than measured for the bulk material. Additionally, we report on a notable effect of the surrounding aqueous glucose solution on the elasticity and the intrinsic viscosity of the studied nanowires made of Ormocomp.

## 1. Introduction

Two-photon polymerization direct laser writing (TPP-DLW) is one of the basic microstructure fabrication techniques used in biomedical and microfluidic applications [1,2,3,4,5]. TPP-DLW is the best choice for producing microstructures of arbitrary shape with spatial resolution on the order of 100 nm and below [6,7]. The mechanical properties of photopolymer micro-objects have been investigated since the very beginning of TPP-DLW use [8]. Early studies focused on the elastic behavior of photopolymer systems. Significant three-orders-of-magnitude differences have been found between the elastic moduli of micro- and/or nano-sized objects, e.g., coiled nanowire springs immersed in different liquids and the corresponding bulk polymer materials [9,10]. Interestingly, the differences diminished when operating similar micro-coils on air or in a vacuum [11]. The same buffer solution effect was observed for micro-cantilevers [12,13]. By contrast, elastic moduli near to bulk values were reported for micro-cantilevers [14] and nanowires [15] operated at dry conditions. These early observations highlighted the importance of buffer/polymer interactions. The elastic moduli size-scaling reported for micro- and nano-sized objects was ambiguous. Depending on the conditions, both increasing [11,16] and decreasing [10,12,13,17] elastic moduli were observed towards smaller object dimensions. As discussed recently by Ladner et al. [16], the competing impacts of enhanced polymer chain alignment and the lower degree of conversion in smaller features may explain the contradictory results.

A variety of applications have been reported that use flexible microstructures prepared by TPP-DLW. For example, the 3D scaffold systems used for cell cultivation are mentioned [18], the deformations of which have been utilized to measure the forces exerted by live cells [19]. Flexible microstructures have been used to perform flow-rate measurements inside microfluidic channels [20] and also to create micro-mechanical logic gates [21]. The systems mentioned so far exploit the static (slow motion) material deformations determined by the material elasticity. Other flexible microstructures were operated in a dynamic regime. Acoustically excited micro-jets and micro-thrusters were prepared by direct laser writing of hydrogels [22]. Alternatively, UV photo-curing was applied to fabricate simple micro-swimmers equipped with a flagellum [23]. The polymer elastic characteristics are not sufficient to treat the dynamic behavior of microstructures. One needs to take the viscoelastic material properties into account.

In this work, we focus on photopolymer nanowires. Different experimental techniques have been developed to characterize the viscoelasticity of macroscopic fibers [24,25]. The process of downscaling experiments to microscopic dimensions is complex. Viscoelastic behavior was reported recently for nanowires prepared by TPP-DLW [26,27]. In the work of Cayll et al., the nanowire properties were measured by a MEMS-based dynamic mechanical analyzer and were expressed in terms of the frequency dependent tensile storage and loss moduli [26]. Viscoelasticity of photopolymer nanowires was also reported in our previous work [27]. The bending recovery motion was studied for cantilevered nanowires equipped with a microbead at their free end and immersed in Newtonian liquids. The microstructures were deflected by an optical tweezer to initiate the motion. A simple viscoelastic mechanical model was proposed for the nanowire to describe the observed double-exponential time dependence of the overdamped bending recovery motion. The nanowire model worked with three parameters: two spring constants and an intrinsic damping coefficient and was used to analyze the characteristic bending recovery times. It is noted that the selected mechanical model described the endpoint deflection of the whole nanowire. The viscoelastic properties of the photopolymer material need to be derived separately; the nanowire shape and dimensions must be considered. Our previous results confirmed the importance of the solution/microstructure interactions; however, the parameters of the mechanical model were not obtained explicitly. This prevented us from proceeding towards the material properties that are targeted in the present work.

The feasibility of the three-parameter approach for describing the viscoelasticity of photopolymer nanowires was also proved in relation to the Brownian fluctuations of the studied microstructures [28]. Relying on the fluctuation–dissipation theorem, the proposed mechanical model was complemented by random forces to explain the stochastic motion of the microbeads attached to viscoelastic nanowires. The double-Lorentzian power spectral density, derived from the model, was well reproduced by experimental observations [28].

In the present work, we build on our previous results and investigate the bending recovery of cantilevered nanowires in more detail. It is our primary goal to provide a simple and useful description of the photopolymer nano-material viscoelasticity. Two necessary steps are made to promote the quantitative analysis of the viscoelastic material properties. First, we show the relations that convert the mechanical model parameters to the photopolymer material properties as defined by the three-parameter standard linear solid model. Second, the theory is extended; relations are derived to calculate the mechanical model parameters using the experimental recovery motion data. Ormocomp, the commercial biocompatible photoresist [29], was used to fabricate the microstructures. The effect of glucose solutions on the mechanics of Ormocomp nanowires was examined in detail.

## 2. Theoretical Considerations

### 2.1. The Three-Parameter Solid Model

**Figure 1:**
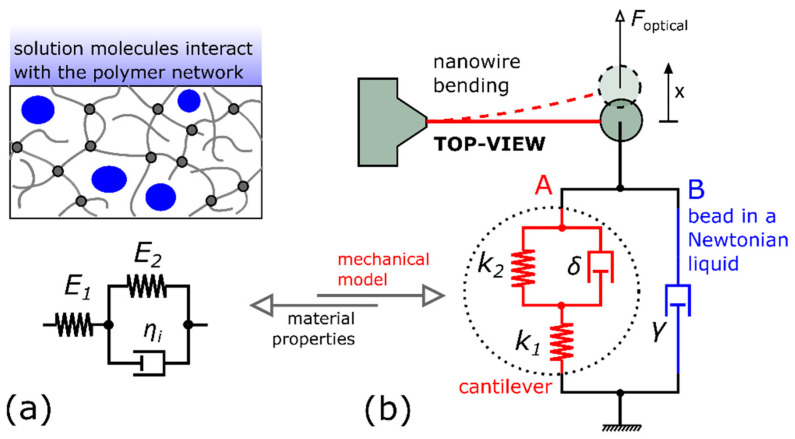
The scheme showing the interconnection of the used models: (**a**) the 3-parameter solid model of the photopolymer material; (**b**) the mechanical model of the cantilever system immersed in a Newtonian liquid.

The nanowire viscoelastic material properties are characterized by the three-parameter solid model (the Kelvin form of “standard linear solid”) depicted in Figure 1a, which contains the combination of two elastic moduli *E*_1_, *E*_2_, and an intrinsic viscosity *η*_i_. The model defines the stress–strain constitutive relation of the photopolymer material. The corresponding creep compliance and relaxation modulus functions can be found in basic textbooks [30]. The standard linear solid model was used previously to characterize the viscoelasticity of macroscopic photopolymer materials [31].

The mechanical properties of thin photopolymer structures are greatly influenced by the conditions near the polymer surface at the solution interface. There is an indication that below a certain thickness, the affected surface layer fills the entire structure. For example, in the case of nanowires made of urethane–acrylate resin, the size-dependent shrinkage was found to saturate below 200 nm thickness [32]. For the sake of simplicity, we assume that the photopolymer nanowires studied here are homogeneous; the possible inhomogeneities across the nanowires are neglected [15]. Consequently, the derived material properties should be considered as effective values, which are representative for the studied thin photopolymer structures.

### 2.2. The Mechanical Model of the Microstructure

The fabricated nanowire cantilever is equipped with a microbead attached to its free end (see Figure 1b) and is immersed in a Newtonian liquid. In the thin nanowire limit, the external viscous damping force of the surrounding liquid acts predominantly on the attached bead. The same applies to the optical trapping force. Consequently, the two external forces driving the cantilever motion are only exerted on the beam endpoint. With these conditions, using the elastic–viscoelastic correspondence theorem [30], we can derive the relations that connect the photopolymer material properties (Figure 1a) to the mechanical model depicted in Figure 1b. The proposed mechanical model already describes the deflection of the cantilever endpoint and the motion of the attached microbead [27]. It is noted that the inertial forces are neglected in the model, which is a reasonable assumption at microscale dimensions [33].

**Figure 2:**
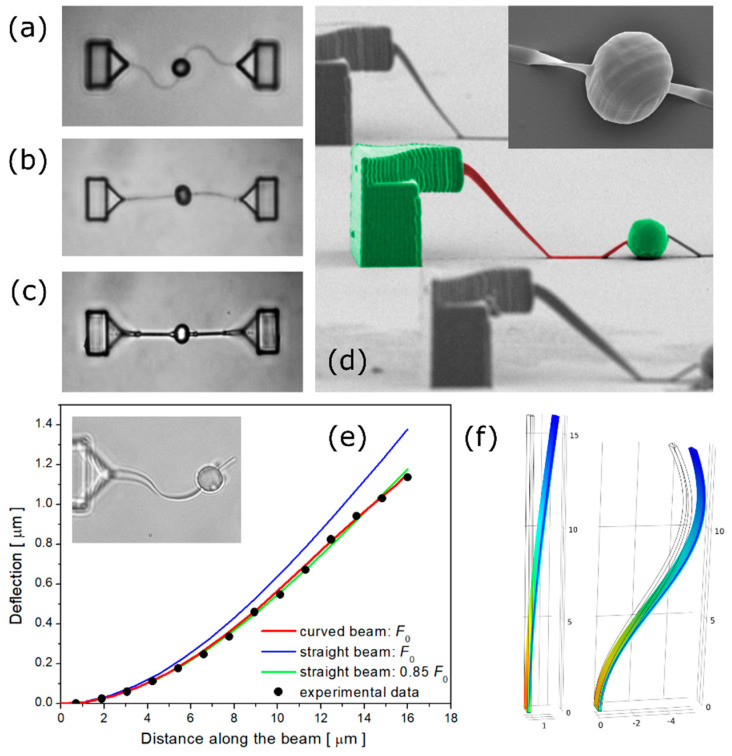
Top-view brightfield images of microstructures immersed in: (**a**) OrmoDev developer; (**b**) water; (**c**) air. (**d**) Side-view SEM image of the dried microstructures, the inset shows the spherical bead. (**e**) Steady-state deflection of the nanowire plotted against the distance along the cantilever beam. The experimental beam deflection (solid points) is compared with the results of straight and curved beam simulations. Inset: the nanowire with the bead immersed in water. (**f**) Deflection of straight and curved cantilevers simulated by finite element method.

The left arm (A) in Figure 1b belongs to the nanowire. In the case of a straight beam, the two spring constants (stiffnesses) *k*_1_ and *k*_2_, and the intrinsic damping coefficient *δ* can be expressed as follows (see the Supplementary Information):

(1) $$k_{1,2}=\frac{3I}{l^{3}}E_{1,2}$$

(2) $$\delta =\frac{3I}{l^{3}}\eta_{i}$$

where *l* is the length of the cantilever and *I* is the second moment of inertia of the beam cross-section. We approximate the flattened nanowire cross-section (see Figure 2d) with a rectangle of sides *a* and *b*, for which *I* = *a*^3^*b*/12.

The mechanical model also contains the damping (or drag) coefficient γ forming the right arm (B) in Figure 1b, which stands for the viscous drag force opposing the motion of the spherical microbead in the Newtonian liquid. The drag coefficient can be estimated from Stokes’ law:

(3) γ =6πηr

where *r* is the sphere radius and *η* represents the liquid viscosity.

### 2.3. The Bending Recovery Motion

The bending recovery of the nanowire microstructure is a unique type of creep experiment. The optical trap is used during the initial phase to induce the cantilever deflection; the nanowire is allowed to equilibrate in the deflected position. In the next step, the optical trap is switched off. As the applied optical force shifts to zero, the nanowire gradually returns to its original equilibrium position. In general, the recovery motion is strongly overdamped. The microbead’s equation of motion during the recovery phase has been solved previously [27]. The time-dependence of the nanowire endpoint deflection *x*(*t*) is described by a double-exponential decay:

(4) $$x\left(t\right)=A_{1}\exp\left(-\frac{t}{\tau_{1}}\right)+A_{2}\exp\left(-\frac{t}{\tau_{2}}\right)$$

The two amplitudes *A*_1,2_ and the two decay times *τ*_1,2_ can be determined from the experiments. Furthermore, these parameters can be calculated when the four model parameters *k*_1,2_, δ, and γ are known [27].

### 2.4. The Inverse Problem

In this work, the inverse problem is solved. As shown in the Supplementary Information (see Equations (S13), (S15), and (S16)), the model parameters of the nanowire cantilever can be expressed in the following form:

(5) $$k_{1}=\gamma \frac{A_{2}\tau_{1}^{2}+A_{1}\tau_{2}^{2}}{\tau_{1}\tau_{2}\left(A_{2}\tau_{1}+A_{1}\tau_{2}\right)}$$

(6) $$k_{2}=\gamma \frac{\left(A_{2}\tau_{1}^{2}+A_{1}\tau_{2}^{2}\right)\left(A_{2}\tau_{1}+A_{1}\tau_{2}\right)}{A_{1}A_{2}\tau_{1}\tau_{2}{\left(\tau_{1}-\tau_{2}\right)}^{2}}$$

(7) $$\delta =\gamma \frac{{\left(A_{2}\tau_{1}^{2}+A_{1}\tau_{2}^{2}\right)}^{2}}{A_{1}A_{2}\tau_{1}\tau_{2}{\left(\tau_{1}-\tau_{2}\right)}^{2}}$$

Using these equations, the stiffnesses *k*_1_ and *k*_2_, and the intrinsic damping coefficient δ can be determined experimentally. Moreover, when combined with Equations (1) and (2), one can measure the material properties of the viscoelastic nanowires, as defined by the standard linear solid model. It is noted that the right sides of Equations (5)–(7) only depend on the ratio of the two amplitudes *A*_1_/*A*_2_. Consequently, the measured cantilever deflection does not require calibration in the experiment. The results are independent of the extent of the initial deflection; relative deflection measurements are sufficient to determine the three model parameters. On the other hand, the damping coefficient γ is not excluded from these formulas and must be calculated separately from Equation (3).

## 3. Materials and Methods

The cantilevered nanowire structures were prepared by TPP-DLW of Ormocomp, the commercial biocompatible photoresist [29]. Ormocomp is a hybrid organic-inorganic photopolymer that belongs to the class of Ormocers (Organically Modified Ceramics) [34]. The average laser power of the 785 nm (100 MHz repetition rate, 100 fs pulse length) laser was set to 6 mW. The laser beam was focused into a photoresist droplet by a 40× oil immersion objective (NA 1.3). The piezo-driven sample stage was maneuvered using pre-programming to write the microstructures. The nanowire, anchored to the vertical support at a height of 8 µm, was drawn as a single line with a 50 µm/s scan speed. The diameter of the attached bead was 5 µm. The system was stabilized by drawing an identical nanowire to the opposite side of the bead (see Figure 2a–c), which was cut and removed before the measurements. The structures were washed in an OrmoDev developer and were irradiated by a microscope mercury lamp (HBO50) to promote post-polymerization. Next, the structures were washed in water and air-dried for storage. As shown in Figure 2a, the nanowires used in the present experiment were polymerized in a curved shape. Pronounced nanowire shrinkage was observed after submerging the microstructures in water (Figure 2b). The curved nanowire shape was required to minimize the risk of fracture during this stage. The shrinkage effect of hydrophobic photopolymers was utilized previously to fabricate self-assembling microfluidic devices [35]. The present nanowires, after being dried, adhered to the bottom glass surface (Figure 2c,d). The width and the height of the dried nanowires were ca. 200 nm and 900 nm, respectively. The original curved shape of the nanowire was recovered when the auxiliary cantilever was removed, and the structure was immersed in water for the measurements (see the inset of Figure 2e). The final arc length of the nanowire was ca. 16 µm.

A holographic optical tweezer setup was used to deflect the cantilevers immersed in aqueous glucose solutions. The power of the 1070 nm laser beam was set to 150 mW at the sample. During the deflection period, the phase pattern applied to the SLM (spatial light modulator) [36] was gradually modified to induce the horizontal motion of the trapped bead. The cantilever deflection angle was kept below 90 mrad. After the initial deflection phase, the structures were allowed to equilibrate in a deflected position for two seconds. Subsequently, the trapping laser beam was switched off and the microbead motion was followed by video tracking. To suppress the effect of Brownian fluctuations [28], the results of twelve repetitive recovery actions were averaged for each glucose concentration.

The nanowires used in the present experiments were of curved shape (see the inset in Figure 2e) while the theory was derived for straight beams. A finite-element-numerical model (prepared with COMSOL Multiphysics) was used to estimate the differences between straight and curved cantilevers of the same arc length (see Figure 2f). In the first step, an elastic curved cantilever model (approximated by a sine shape) was tuned to reproduce the experimental steady-state cantilever deflection induced by the optical force (Figure 2e, solid points). The observed deflection distribution along the cantilever beam was successfully reproduced by the model (see Figure 2e, red curve). In the next step, an identical force *F*_0_ was applied to the endpoint of a straight beam. The obtained deflection was about 20% higher in this case (the blue curve in Figure 2e). The experimental deflection curve could be reproduced by lowering the applied force to 0.85 times *F*_0_ (see the green curve in Figure 2e). After this force correction, the general shape of the deflection distribution was almost identical for the straight and curved beams. It was concluded that accounting for the proposed force correction, the straight beam theory, and the corresponding Equations (1) and (2), represented an accurate approximation for the studied curved nanowire system.

## 4. Results and Discussion

**Figure 3:**
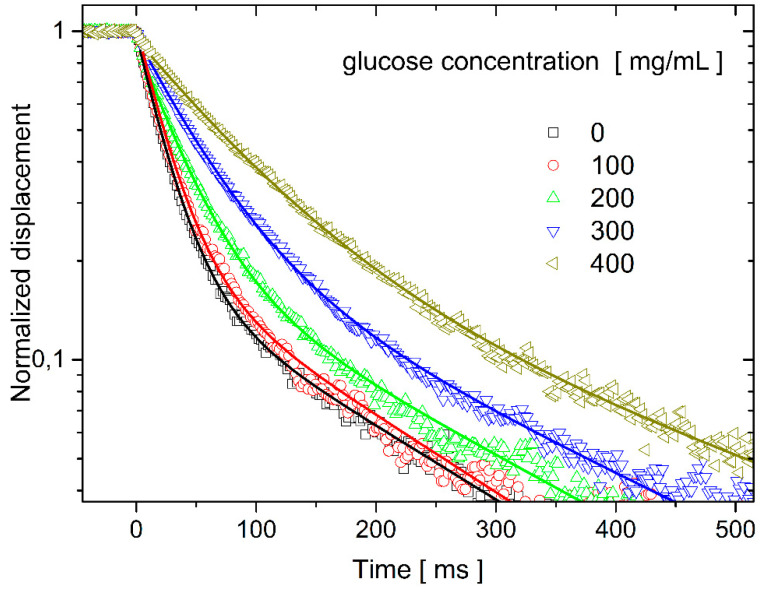
The experimental bending recovery curves of the cantilever nanowire system were measured at different glucose concentrations. The data points were fitted by double-exponential time dependencies (solid lines).

The nanowire bending recovery motion was followed in different glucose solutions. The glucose concentration was varied between 0 and 400 mg/mL. Selected normalized recovery curves are shown in Figure 3. The experimental data points are well fitted with double-exponential decays, as predicted by the mechanical model (see Equation (4)). In general, the overdamped recovery motion slows down as the viscosity of the surrounding solution increases.

Detailed data analysis was performed in accordance with the present theory. In the first step, the characteristic decay times *τ*_1,2_ and the corresponding amplitudes *A*_1,2_ were used to calculate the stiffnesses *k*_1,2_ and the intrinsic damping coefficient δ, as defined by Equations (5)–(7). Representative results, obtained for pure water and 400 mg/mL glucose solution, are presented in Table 1. The equilibrium stiffness *k*_eq_ of the cantilever, also included in Table 1, is obtained as a combination of the two spring constants: 1/*k*_eq_ = 1/*k*_1_ + 1/*k*_2_. This value refers to the static response of the nanowire and determines its strength in the slow-motion limit. Interestingly, the obtained equilibrium stiffness is in the order of 0.002 pN/nm, which proves the extreme flexibility (softness) of the studied nanowires and points towards possible future applications in the field of sensitive force measurements. The intrinsic damping δ of the studied cantilever can be compared with the drag coefficient γ for the bead motion in the surrounding medium (Table 1). Notably, for the present experimental conditions, the intrinsic damping (as defined by the mechanical model), is more than an order of magnitude higher than the drag coefficient of the solution acting on the microsphere.

**Figure 4:**
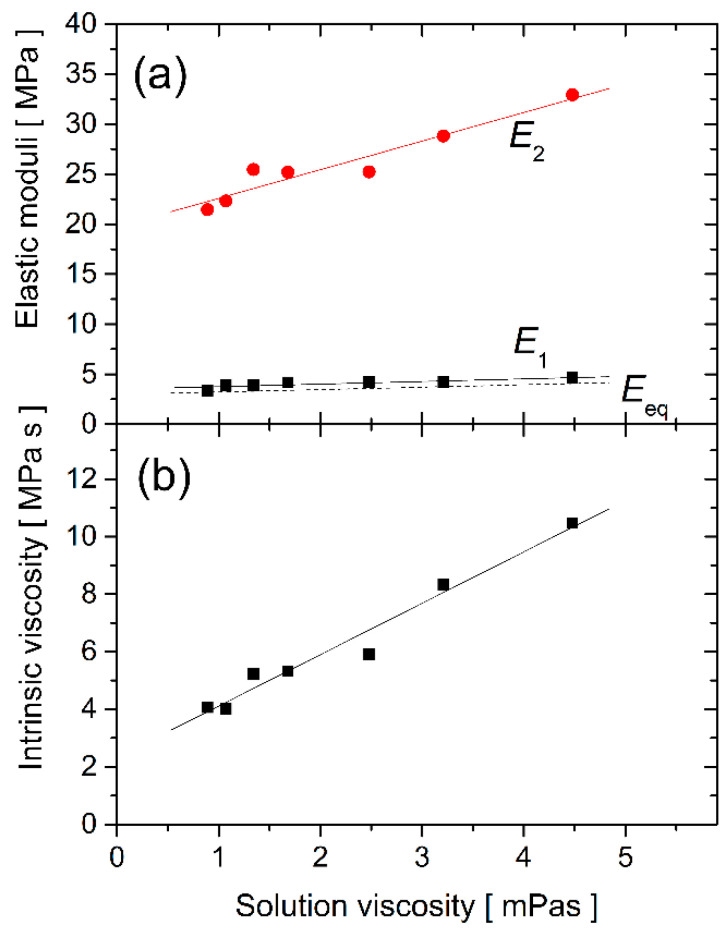
The nanowire material properties: the elastic moduli *E*_1_, *E*_2_, and *E*_eq_ (**a**), and the intrinsic viscosity *η*_i_ (**b**), are plotted against the glucose solution viscosity.

The experimental results discussed characterize the specific microstructure studied. The corresponding material properties of the nanowire can be estimated from Equations (1) and (2). The obtained elastic moduli *E*_1_, *E*_2_, and the intrinsic viscosity *η*_i_ are plotted in Figure 4 as a function of the solution viscosity. Selected parameter values are given in Table 1. The equilibrium elastic modulus *E*_eq_, defined as: 1/*E*_eq_ = 1/*E*_1_ + 1/*E*_2_, was also calculated and is plotted in Figure 4a by the dashed line. The nanowire elastic moduli can be analyzed in terms of the bulk Young modulus, which is approximately *E*_bulk_ = 1 GPa (producer data, [29]). For the studied conditions, the measured equilibrium elastic modulus of the nanowire is about 300 times smaller (*E*_eq_ = 3.5 ± 0.6 MPa, see Table 1). Significantly reduced elastic moduli of nano- and micro-structures prepared by TPP-DLW were observed for other photopolymers immersed in different solutions [9,10,12]. These previous measurements were performed at static conditions. The present dynamic studies, analyzed by the three-parameter solid model, provide a more nuanced representation of the material. The equilibrium modulus *E*_eq_ is determined predominantly by the *E*_1_ values, which are substantially lower than *E*_2_.

At present, there is no definite relation between the model parameters and the nanowire molecular structure; different models can be used to reproduce the same experimental data. Regardless, the present model indicates a strong interaction between the used photopolymer material and the glucose content of water. The *E*_2_ elastic modulus increases towards higher glucose concentrations. We observe the same tendency for *E*_1_ and *E*_eq_. These data indicate that glucose molecules stiffen the nanowires made of Ormocomp.

The effect of glucose is further pronounced for the intrinsic viscosity plotted in Figure 4b. The intrinsic viscosity of the polymer material is as high as 4 MPas, even for pure water conditions. This indicates a marked portion of uncured polymer material inside the nanowire structure and is likely related to the limited degree of conversion observed for TPP-DLW structures near the polymerization threshold [10,15,37,38]. Moreover, the value of *η*_i_ changes from 4.1 to 10 MPas, when the surrounding solution viscosity is varied between 0.9 and 4.5 mPas. There must be a high affinity of glucose molecules to the porous polymer structure. The prolonged time, ca. 15–20 min, needed to restore the mechanical properties of the nanowire cantilever when moving back from glucose solutions to pure water (not shown) supports this assumption. A similar effect was observed recently for commercial polyurethane foam immersed in dextran solutions [39]. It would be desirable to study the Ormocomp–glucose interaction in more detail, focusing on the solvent permeability and the glucose penetration depth, which is beyond the scope of the present work.

The results presented in Figure 4 rely on Equations (1) and (2). It was assumed that the nanowire size did not change significantly in different buffer solutions. Indeed, the analysis of the bright-field images taken at different glucose concentrations only showed a minor prolongation of the nanowire (on the order of 1%) when 400 mg/mL glucose was added to water. These low-level size changes may not be the reason for the pronounced glucose concentration dependence observed in Figure 4.

## 5. Conclusions

The viscoelasticity of photopolymer nanowires prepared by TPP-DLW of Ormocomp was studied, employing bending recovery experiments performed on a cantilevered nanowire system. The observed bending recovery curves show a double-exponential time-dependence, which aligns with the assumption that the nanowire viscoelasticity can be described by the standard linear solid model. The corresponding three material parameters, two elastic moduli, and intrinsic viscosity are evaluated by analyzing the experimental data. The results indicate a significantly reduced stiffness of the nanowire polymer structure as compared to the bulk conditions. Additionally, a pronounced effect of the buffer solution is observed. The presence of glucose in the solution enhances both the elastic moduli and the intrinsic viscosity of the polymer network. It is hypothesized that the concept of polymer network permeability to solvents, as proposed by Takada et al. [32], and the affinity of glucose to Ormocomp explain this phenomenon. Further experiments are needed to verify the universal nature of the present results when working with other photopolymer materials and/or other buffer solutions.

## Figures and Tables

**Table 1:** Representative values of the mechanical model parameters (stiffnesses *k*_1,2_ and the intrinsic damping coefficient *δ*) and the corresponding material properties (elastic moduli *E*_1,2_, and the intrinsic viscosity *η*_i_) are evaluated for pure water and dense glucose solution conditions. The equilibrium stiffness *k*_eq_ is calculated as: 1/*k*_eq_ = 1/*k*_1_ + 1/*k*_2_. The equilibrium elastic modulus *E*_eq_ is obtain analogously.

	*k*_1_ pN/nm	*k*_2_ pN/nm	*k*_eq_ pN/nm	*δ* 10^−6^ kg s^−1^	γ 10^−6^ kg s^−1^	*E*_1_ MPa	*E*_2_ MPa	*E*_eq_ MPa	*η*_i_ MPa s
water	0.0017	0.011	0.0015	2.1	0.042	3.4	21	2.9	4.1
400 mg/mL glucose	0.0024	0.017	0.0021	5.4	0.21	4.6	33	4.1	10

## Supplementary Materials

The following are available online at https://www.mdpi.com/article/10.3390/nano11112961/s1, The theoretical background of Equations (1), (2) and (5)–(7).
