# Supplementary material for: Assessing the Viscoelasticity of Photopolymer Nanowires Using a Three-Parameter Solid Model for Bending Recovery Motion
Source: Nanomaterials (Basel). 2021 Nov 4;11(11):2961. doi: 10.3390/nano11112961 (PMC8618069; doi:10.3390/nano11112961)
Supplement: Supplementary file 1 [file nanomaterials-11-02961-s001.zip › nanomaterials-1411022-supplementary.pdf]

Supplementary Material: Assessing the viscoelasticity of photopolymer nanowires using a three-parameter solid model for bending recovery motion

Jana Kubacková, Cyril Slabý, Denis Horvath, Andrej Hovan,
Gergely T. Iványi, Gaszton Vizsnyiczai, Lóránd Kelemen,
Gabriel Žoldák, Zoltán Tomori, Gregor Bánó

Supplementary material is divided into two separate parts, two sections. The first one deals with the context and the general *correspondence principle* that links the mechanical model and the material parameters. The second focuses on a specific measurement methodology which provides k_1, k_2 and δ mediated by a double exponential deflection in a time-dependent bending recovery experiments.

1 The mechanical model connection with material parameters

The relations that link the photopolymer material properties to the mechanical model (Figure 1 in the main text) can be determined on the basis of the elastic-viscoelastic *correspondence theorem* [1]. The deflection of a viscoleastic beam subject to a load applied at $t = 0$ and then held constant is derived from the deflection of the corresponding elastic beam by replacing the reciprocal Young's modulus $1/E$ [1] by the time-dependent creep-compliance function $J(t)$. Assume that the lateral force F_e (beginning at $t = 0$) applies just on the endpoint of the cantilevered nanowire in air, that is, without any relevant surrounding medium. We note that this procedure applies only to this section and is illustrated here by a thought experiment to derive the relationship between $k_{1,2}$ and $E_{1,2}$, and between δ and η_i.

For purely elastic conditions, Euler's beam theory gives the end-point deflection x_e in a form

$$x_\mathrm{e} = \left(\frac{F_\mathrm{e} l^3}{3I}\right) \frac{1}{E}\,. \tag{S1}$$

Here, l is the length of cantilever, I is the second moment of inertia of the beam cross section. The creep-compliance of the standard linear solid in the Kelvin's formulation [1] (see Fig.1a in the main text) is given by

$$J(t) = \frac{1}{E_1} + \frac{1}{E_2} - \frac{1}{E_2} \exp\left(-\frac{E_2 t}{\eta_i}\right)\,. \tag{S2}$$

Substituting $J(t)$ for $1/E$ in Eq.(S1) we obtain single exponential temporal behavior of the viscoelas-

[1] What we are referring here is newly introduced E, which belongs to the purely elastic structure.

tic beam deflection

$$x_{\mathrm{ve}}(t) = \left(\frac{F_{\mathrm{e}} l^3}{3I}\right) \underbrace{\left[\frac{1}{E_1} + \frac{1}{E_2} - \frac{1}{E_2}\exp\left(-\frac{E_2 t}{\eta_i}\right)\right]}_{=J(t)} \tag{S3}$$

in this special case. We may also extract the time dependent deflection of the beam endpoint

$$x_{\mathrm{ve}}(t) = F_{\mathrm{e}}\left[\frac{1}{k_1} + \frac{1}{k_2} - \frac{1}{k_2}\exp\left(-\frac{k_2 t}{\delta}\right)\right] \tag{S4}$$

represented in terms of k_1, k_2, and δ by solving the mechanical model of the viscoelastic cantilever for the identical load conditions. A direct comparison of Eqs.(S3) and (S4) leads to $k_{1,2} = 3(I/l^3)E_{1,2}$ and $\delta = 3(I/l^3)\eta_i$ presented in equations (1) and (2) in the main text of the paper.

2 Double exponential decay basis for a measurable deflection

We continue with a summary of previously published derivations [2] and then move on to supplementary derivations of formulas for the mechanical model parameters. The nanovire elasticity is explained in the mechanical model framework by the parameters k_1, k_2. Both the damping coefficient δ, and the hydrodynamic drag γ are associated to dissipative processes. The final objective of the supplementary material is to establish a methodological relationship between theoretical model parameters k_1, k_2, δ and the bending recovery parameters measured or estimated by a regression (A_1, A_2, τ_1, τ_2; see below).

Assume now that the location of the microsphere on the deflection trajectory is described by the dynamic coordinate $x(t)$. Neglecting the inertial forces, we obtain a system of two first order differential equations, the combination of which yields a single second-order differential equation of the form

$$\left(\delta\gamma\frac{\mathrm{d}^2}{\mathrm{d}t^2} + [\,k_1(\delta+\gamma) + k_2\gamma]\,\frac{\mathrm{d}}{\mathrm{d}t} + k_1 k_2\right) x(t) = 0\,, \tag{S5}$$

which is formulated in terms of suitable initial conditions $\mathrm{d}^2x/\mathrm{d}t^2\big|_{t=0}$ and $\mathrm{d}x/\mathrm{d}t\big|_{t=0}$. These conditions must take into account the fact that the optical forces are maintaining the cantilever at a constant deflection before the recovery process starts ($t = 0$). The solution can be determined by the characteristic equation

$$\left(\frac{1}{\tau}\right)^2 \delta\gamma - \left(\frac{1}{\tau}\right)[\,k_1(\delta+\gamma) + k_2\gamma\,] + k_1 k_2 = 0\,. \tag{S6}$$

It is used to represent the system, and its eigenvalues $(1/\tau)_{1,2}$ to provide a pair of the relaxation times

$$\frac{1}{\tau_{1,2}} = \frac{1}{2\delta\gamma}\left[\,k_1(\delta+\gamma) + k_2\gamma \pm \sqrt{[\,k_1(\delta+\gamma)+k_2\gamma\,]^2 - 4k_1k_2\delta\gamma}\,\right]\,. \tag{S7}$$

This suggests that fundamental system of solutions exists $\{\exp(-t/\tau_1),\ \exp(-t/\tau_2)\}$, with the possibility of constructing a superpositioned double-exponential decay $x(t) = A_1\exp(-t/\tau_1) + A_2\exp(-t/\tau_2)$ if the discriminant is positive and $\tau_{1,2} > 0$). The properties of relaxation times

$$\frac{1}{\tau_1\tau_2} = \frac{k_1 k_2}{\delta\gamma}\,, \tag{S8}$$

$$\frac{1}{\tau_1} + \frac{1}{\tau_2} = \frac{k_1(\delta+\gamma) + k_2\gamma}{\delta\gamma} \tag{S9}$$

can be deduced easily. From Eq.(S8) we simply have

$$\delta = \frac{k_1 k_2}{\gamma} \tau_1 \tau_2 \ . \tag{S10}$$

After substituting into Eq.(S9) we get

$$\tau_1 + \tau_2 - \frac{k_1}{\gamma} \tau_1 \tau_2 = \gamma \frac{k_1 + k_2}{k_1 k_2} \ . \tag{S11}$$

The right-hand side of the obtained connection has a more profound interpretation, which is linked to the component of weighted average time that most of us provided and validated in our earlier work [2]. As a result, we obtain the expression

$$\tau_{\mathrm{e}} = \gamma \frac{k_1 + k_2}{k_1 k_2} = \frac{\tau_1 \tau_2 (A_1 + A_2)}{A_1 \tau_2 + A_2 \tau_1} \, . \tag{S12}$$

Here we reintroduce τ_{e} as a distinguishable component of the weighted average time proportional to γ. Combining Eq.(S11) and Eq.(S12) we obtain

$$k_1 = \frac{\gamma}{\tau_1 \tau_2} \left(\tau_1 + \tau_2 - \tau_{\mathrm{e}} \right) = \gamma \frac{A_2 \tau_1^2 + A_1 \tau_2^2}{\tau_1 \tau_2 (A_2 \tau_1 + A_1 \tau_2)} \, . \tag{S13}$$

Then, using Eq.(S11), we get first k_2 as a function of k_1 in the form

$$k_2 = \frac{k_1}{\frac{k_1(\tau_1 + \tau_2)}{\gamma} - \frac{k_1^2 \tau_1 \tau_2}{\gamma^2} - 1} \ . \tag{S14}$$

After that by substituting k_1 we get

$$k_2 = \gamma \frac{(A_2 \tau_1^2 + A_1 \tau_2^2)(A_2 \tau_1 + A_1 \tau_2)}{A_1 A_2 \tau_1 \tau_2 (\tau_1 - \tau_2)^2} \, . \tag{S15}$$

Finally, we return to the formula for δ [see Eq.(S10)] and derive the expression

$$\delta = \gamma \frac{(A_2 \tau_1^2 + A_1 \tau_2^2)^2}{A_1 A_2 \tau_1 \tau_2 (\tau_1 - \tau_2)^2} \tag{S16}$$

by inserting k_1, k_2 from Eqs.(S15) and (S13).

References

[1] W. Flügge, Viscoelasticity (Springer-Verlag Berlin Heidelberg GmbH, 1975).

[2] J. Kubackova, G. T. Ivanyi, V. Kazikova, A. Strejckova, A. Hovan, G. Zoldak, G. Vizsnyiczai, L. Kelemen, Z. Tomori, and G. Bano. "Bending dynamics of viscoelastic photopolymer nanowires." Appl. Phys. Lett. vol. 117, 013701, 2020.
